# Contribution of Neurons and Glial Cells to Complement-Mediated Synapse Removal during Development, Aging and in Alzheimer's Disease

**DOI:** 10.1155/2018/2530414

**Published:** 2018-11-11

**Authors:** Celia Luchena, Jone Zuazo-Ibarra, Elena Alberdi, Carlos Matute, Estibaliz Capetillo-Zarate

**Affiliations:** ^1^Achucarro Basque Center for Neuroscience, CIBERNED and Departamento de Neurociencias de la Universidad del País Vasco UPV/EHU, E-48940 Leioa, Spain; ^2^IKERBASQUE, Basque Foundation for Science, 48011 Bilbao, Spain; ^3^Weill Cornell Medical College, Cornell University, New York, NY 10065, USA

## Abstract

Synapse loss is an early manifestation of pathology in Alzheimer's disease (AD) and is currently the best correlate to cognitive decline. Microglial cells are involved in synapse pruning during development via the complement pathway. Moreover, recent evidence points towards a key role played by glial cells in synapse loss during AD. However, further contribution of glial cells and the role of neurons to synapse pathology in AD remain not well understood. This review is aimed at comprehensively reporting the source and/or cellular localization in the CNS—in microglia, astrocytes, or neurons—of the triggering components (C1q, C3) of the classical complement pathway involved in synapse pruning in development, adulthood, and AD.

## 1. Introduction

Alzheimer's disease (AD) is a neurodegenerative disorder, which is clinically characterized by progressive cognitive decline finally leading to the full-blown picture of dementia [1]. AD represents 50 to 70% of all dementia cases. Yet, it has no cure. Synaptic loss and dendritic loss have been observed in the hippocampus and neocortex of AD patients [2]. The decrease in cortical synaptic density is mirrored by changes in the presynaptic marker synaptophysin and correlates with cognitive decline in AD patients [3]. Therefore, understanding the underlying mechanisms responsible for synapse loss during AD is of critical importance in order to identify new therapeutic targets.

The complement system is part of the innate immune system in multicellular organisms, and it is activated by three biochemical pathways (explained in more detail in Section 2). The classical complement pathway is activated when ligands bind to C1q triggering C1 complex activation. C3, a central protein of the complement cascade, acts as downstream of C1q in the classical complement cascade and also activates the alternative pathway when ligands bind directly to it. Recent publications point towards a role played by microglia and astrocytes in early synapse pruning during development, presumably via the classical complement pathway [4]. They also showed that expression of C1q protein by retinal neurons modulated by astrocytes was a crucial event for synaptic pruning [5].

In AD, complement components have been associated with amyloid-*β* (A*β*) plaques [6, 7]. It has also been reported that oligomeric/fibrillar A*β* and hyperphosphorylated tau (pTau) activate the complement pathway by binding to C1q [8–13]. C1q is upregulated and associated to synapses in the presence of oligomeric A*β* [8]. Under these circumstances, the classical complement pathway activates and results in synapse loss before A*β* deposition takes place [8]. C3 has been localized on reactive astrocytes in human AD cases [14] and they might contribute to synapse loss by releasing complement components themselves.

The origin and contribution of complement proteins to synapse pruning in development and AD are not well understood and require further investigation. The present review is aimed at addressing the role of microglia and astrocytes in complement-mediated synaptic pruning associated with development, adulthood, aging, and AD.

## 2. Complement-Mediated Synapse Pruning during Development

During development, active synapses mature, while less active ones are engulfed and removed by microglia [15–18]. Synapse removal is regulated, among other mechanisms, by the classical complement cascade.

The complement system can be activated by three different pathways: (1) the classical pathway commonly is initiated by antigen-antibody binding leading to the phagocytosis and/or pore formation in membrane, lysis, and cell death, (2) the alternative pathway is continuously active at low levels and not activated by pathogen or antibody binding. It leads to opsonization and kills pathogens, and (3) the lectin pathways are activated by mannan-binding lectin as opposed to antibody-antigen recognition (reviewed in [19]). In this review, we will focus on the classical pathway. This pathway is initiated when the antigen-antibody complex binds to the C1q protein. In addition to antigen antibody activation of C1q, antibody-independent activation of C1q has been consistently described [9, 10, 20–22]. C1q protein is the recognition subcomponent of the C1 complex, which is composed of one molecule of C1q, two molecules of C1r, and two molecules of C1s (C1qr^2^s^2^). C1q recognizing and binding to different ligands trigger the cascade of events leading to the cleavage of complement component C3 into the fragments C3a and C3b. It is worth mentioning that the cleavage of C3 can also be directly activated by the alternative pathway. C3b is one of the primary complement opsonins and has been shown to tag synapses targeted for elimination [15, 19, 23]. Further C3b cleavage gives rise to iC3b, which subsequently binds to complement receptor 3 (CR3) in microglia, in turn driving a response that, among others, promotes phagocytosis of cellular structures like synapses [24, 25]. Therefore, the cellular localization of C1q and C3 and their receptors is crucial in understanding the mechanism regulating synapse removal.

**Figure 1:**
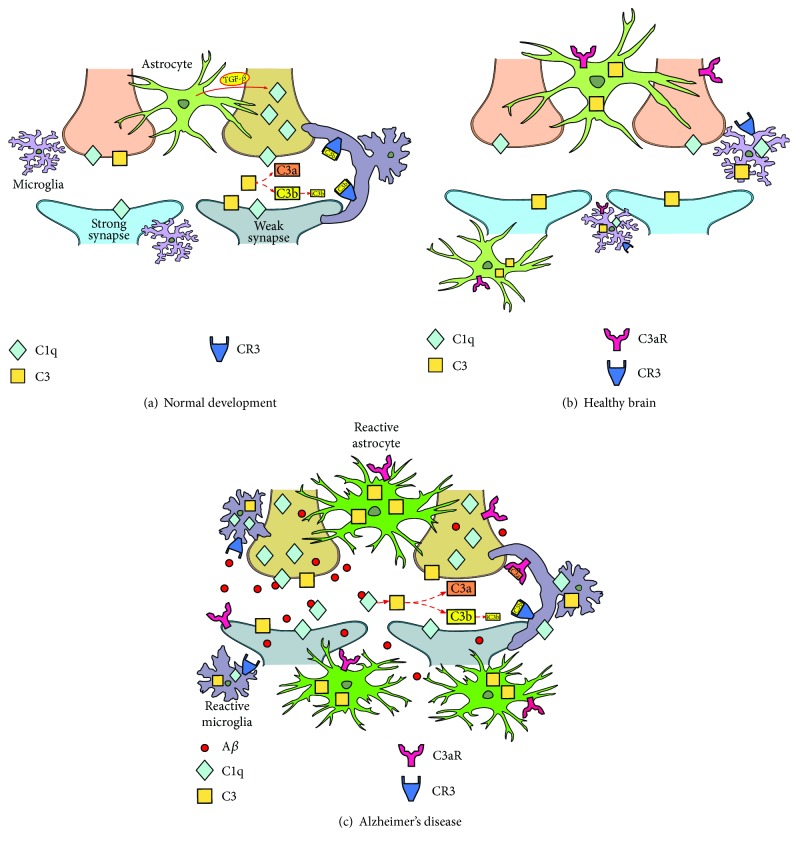
Model of complement-mediated synapse elimination during development, adulthood, and Alzheimer's disease. (a) During early postnatal development, synaptic pruning takes place in order to eliminate excessive or weak synapses. Astrocytes induce the expression of C1q in neurons through TGF-*β*, and C1q colocalizes with synapses. The complement protein C3, which also colocalizes with synaptic puncta, is enzymatically cleaved to smaller fragments C3a and C3b. Finally, microglia engulf the synapse through the interaction of iC3b, the cleavage product of C3b, with its CR3 receptors. (b) In the healthy brain, synaptic pruning decreases with age to basal levels and complement protein expression is reduced. Nonetheless, microglia and astrocytes continuously survey surrounding synapses. (c) AD brain is characterized by progressive accumulation of extracellular and intracellular A*β*, gliosis, and neuroinflammation. Some studies have reported the role of microglia and complement pathway on synapse loss in AD models. Neuron-derived C1q and microglia-derived C1q are recruited to synapses and interact with A*β*. This triggers the activation of complement protein C3, expressed by both astrocytes and microglia. C3 is cleaved to smaller fragments such as C3b and iC3b that tag synapses and bind to CR3 on microglia. All these events lead to the removal of tagged synapses by the latter.

*In vitro* and *in vivo* studies involving the use of mRNA expression and immunohistochemistry techniques (Table 1) have described the localization of C1q in neurons, both in synaptic puncta [4, 5] and axons [5] during development. Also, astrocyte-secreted transforming growth factor-*β* (TGF-*β*) has been demonstrated to increase C1q expression in neurons (Figure 1(a)) [5].

The expression of C3 protein has been shown to be crucial in promoting synapse phagocytosis [24]. However, the cellular source of C3 in the CNS during development remains elusive. Immunohistochemical analysis revealed that C3 is present in synaptic puncta in wild-type mice, whereas C3 knock-out (KO) mice were devoid of it [4, 15]. And, as stated above, this led to proposing that C3 cleavage products (C3b/iC3b) could be tagging synapses for pruning [15].

During development, binding of C3 cleavage products to CR3 promotes phagocytosis. CR3, also called CD11b/CD18 or mac-1, is a receptor for iC3b [26] and is uniquely expressed by microglia in the CNS [15, 27–29]. Removal of CR3 results in increased number of synapses in CR3 KO mice [15].

Altogether, these data suggest that during the developmental period of synaptic refinement, astrocyte-secreted TGF-*β* increases C1q expression in neurons. This results in the release of C3. The ligand is subsequently cleaved to yield C3b/iC3b fragments, which in turn bind to CR3 in microglia ultimately promoting the engulfment of synapses (Figure 1(a)).

## 3. Synapse Pruning in Adulthood and Normal Aging

Although synapse remodeling is described to be persistent during life, once overproduced synapses have been removed during postnatal development, synapse elimination is downregulated and remains stable (Figure 1(b)). In adulthood and under healthy conditions, there is a reduction in the expression of C1q and C3 components [24].

A number of *in vitro* and *in vivo* studies reported that microglial cells are the main source of C1q in adulthood and normal aging (Table 2) [30–32]. Importantly, Linnartz and collaborators showed that sialic acid, a cellular membrane component, was crucial in preventing C1q binding to target molecules. The study demonstrated that desialylated neuronal structures are marked by the complement and cleared by microglia, in a process involving CR3 [31]. This mechanism has been related to neurites [31]. Whether this mechanism is also involved directly in synapse removal needs to be investigated. Even though the main source of C1q in adulthood is microglia, C1q has also been localized at synaptic puncta in human and mouse tissue by immunohistochemistry [32]. Furthermore, positive immunoreactivity of C1q has been observed in a subset of interneurons [32, 33]. In a separate study, astrocyte-derived exosomes obtained from human subjects showed expression of C1q [34].

Both microglia [30, 31, 35] and astrocytes [35, 36] have been reported as primary sources of C3 in primary cultures [30, 35]. Similar to C1q, C3 has also been detected in astrocyte-derived exosomes from human subjects [34]. As indicated before, C3 cleaves into different products including C3a and C3b. C3b cleavage product iC3b binds to CR3 [24, 25], whereas C3a binds to C3aR [14].

As described in Section 2, microglial cells are the only source of CR3 in the brain [37]. Linnartz et al. [31] reported that removal of neurites could be mediated by CR3 under some circumstances as indicated before.

Finally, C3a binds to C3aR, a receptor present in neurons, microglia, and astrocytes [37–42]. C3a is an anaphylatoxin linked to proinflammatory signaling and chemotaxis [25, 43]. In the CNS, C3a-C3aR binding has been reported to mediate synaptic plasticity [14, 43] and microglial phagocytosis [14].

## 4. AD, Synapse Pathology, and Gliosis

The neuropathology associated with AD includes synapse loss, neuronal loss, neurofibrillary tangles (NFT), A*β* accumulation, and gliosis [44]. Neuritic senile plaques and NFT are the two main pathological hallmarks of AD. It is widely known that neuritic plaques are surrounded by dystrophic neurites and activated microglia and astrocytes. Dystrophic neurites appear early in the disease and are considered to be aberrant axon/presynaptic terminals caused by (at least in part) alterations in cytoskeleton and axonal flux [45–48]. NFT are intraneuronal aggregates of pTau protein [49, 50], and NFT deposition correlates with progression of the disease [51, 52].

A*β*, the main component of plaques, is the result of the cleavage of amyloid precursor protein (APP) and accumulates both intra- [53–56] and extracellularly [57, 58] leading to synaptic dysfunction, which is currently the best correlate of cognitive decline in patients [3]. Synaptic dysfunction is reported as an early manifestation of AD [59]. Loss of synaptophysin has been shown to correlate well with A*β* accumulation [60, 61]. *In vitro* studies in primary neuronal cultures from AD mouse model Tg2576 showed that A*β* produced by proteolytic cleavage of mutant SwedishAPP is sufficient to induce synapse pathology [62], demonstrating that neurons have a cell-autonomous mechanism for synapse removal.

A*β* aggregation and accumulation associated with AD pathogenesis trigger an inflammatory response in affected areas of the brain. Thus, active microglia and astrocytes are conspicuous around neuritic plaques [63, 64]. Both microglia and astrocytes modify their morphology to adopt a reactive morphology and undergo functional changes (reviewed in [65–69]). Chronic inflammation states, characterized by sustained reactive gliosis, have been shown to worsen the AD pathology (reviewed in [66]). In animal models of AD, activated microglia undergo a change in morphology, from a typical ramified structure to a more amoeboid morphology. This has been associated with increased proliferation [70, 71] and expression of inflammatory markers [70, 72]. Recently, in AD brain tissue samples, region-specific microglial deterioration associated with decrease in amount of microglia has been described indicating possible differential microglial responses between AD models and patients [68, 73]. Reactive astrocytic processes penetrate A*β* deposit, fragmenting and isolating plaques from the surrounding neuropil [63]. Additionally, reactive glia derived from neurological disorders have been characterized using transcriptomic profiling studies [68, 73]. Disease models suggest the existence of expression of specific genetic profiles for “disease-associated microglia” (DAM) [68, 74, 75].

Recently, activated glial cells have been linked to synapse loss in AD. Activated microglia modulate synapse loss via the complement pathway in an AD model [8]. Other studies have shown that apolipoprotein E (APOE) isoforms control for C1q accumulation in the brain and modulate phagocytosis by astrocytes [76]. APOE4, the major genetic risk factor for the late onset AD, negatively affects the rate of synapse pruning and turnover by astrocytes. C1q protein was significantly increased in the hippocampus of APOE4 knock-in (KI) mice when compared with APOE3 KI mice [76]. Moreover, genome-wide association studies (GWAS) and a network-based integrative analysis have linked genes of the immune system—like complement CR1—with increased risk of developing AD [77, 78].

Despite of neuron-autonomous and A*β*-induced synapse turnover, a number of studies demonstrate that glial cells are also associated with synapse loss in AD. The complement system might play an important role in such a process.

## 5. Complement and Synapse Pathology in AD

Components of the classical complement pathway have been associated with senile plaques [79, 80], fibrillar A*β*, NFT, and dystrophic neurites [11, 63, 64, 81–85]. Oligomeric/fibrillar A*β* and pTau bind to C1q and activate the complement classical pathway [8–13].

Complement components have been studied as possible biomarkers for AD. Daborg et al. [86] found increased C3 levels in AD patients compared with patients suffering from mild cognitive impairment but not progressing to AD. They also showed elevated cerebrospinal fluid (CSF) CR1 levels in mild cognitive impairment that progressed to AD and AD patients when these groups merged. Bonham et al. [87] showed a significant interaction between APOE4 and CSF C3 on both CSF A*β* and CSF pTau. Their results also indicated that A*β* mediates C3 effect on pTau.

Schaffer et al. [15] demonstrated the key role played by microglia in developmental synaptic pruning. Since then, glial cells and the complement pathway have become increasingly relevant in the study of synapse loss associated with AD.

Recently, a study demonstrated the involvement of microglia in synapse pathology at early stages of AD, preceding plaque formation [8], thus supporting the existence of a mechanism described during development and also modulating early pathological conditions during AD.

In AD brains, C1q has been associated with oligomeric/fibrillar A*β* and pTau, and described within neurons and activated-microglia around plaques (Table 3; Figure 1(c)) [9–13, 88]. It was not until recently that C1q was found associated with synapse pathology in AD. C1q expression was increased in a region-specific manner in one-month-old J20 AD mice [8]. A significant increase in C1q and PSD-95 colocalization was also observed in J20 mice and also in wild-type (WT) mice when injected with A*β* oligomers. Subsequently, at 3 months of age, a reduction of postsynaptic marker PSD-95 and synaptic puncta was described in these AD mice. *In vitro* and *in vivo* experiments also associated oligomeric A*β* and C1q with synaptic dysfunction and in a common pathway that resulted in synapse removal [8]. These findings supported previously reported data indicating that oligomeric—but not monomeric—A*β* activates the complement cascade in AD [10, 12, 13] and showed that C1q is one of the key players for synapse loss in early preplaque AD.

Both microglia and astrocytes have been shown as sources of C1q in AD. Microglial C1qa upregulation was reported using fluorescence in situ hybridization techniques in the hippocampi of both J20 and A*β* oligomer-treated WT mice [8]. Additionally, although human AD-cultured astrocytes showed low levels of C1q [84], astrocyte-derived exosomes from human AD showed higher expression of C1q compared to control cases [34]. Furthermore, astrocytic cultures from aged 5xFAD mice showed an increase of C1q expression compared with those from control mice [89]. Such increase was also associated with A*β* plaques. Nevertheless, the association between C1q expression and synapse loss observed in the 5xFAD model requires further clarification.

As described for C1q, C3 has also been associated with synaptic puncta in AD mice [8]. A significant increase of C3 and PSD-95 colocalization was observed in the APP/PS1-AD model. Deletion of C3 in APP/PS1 mice prevented hippocampal synapse loss in 4-month-old APP/PS1 mice [8], supporting the key role played by the C3 complement component in synapse loss in AD at early stages of the disease.

A study has reported that exposure to A*β* activates astroglial NF*κ*B, resulting in the release of C3 by astrocytes. This in turn leads to reduced synaptic density and altered dendritic morphology [14]. C3 cleavage products including C3a and C3b have been shown to mediate A*β* phagocytosis under pathological conditions [43, 90].

In AD, as showed in development and adulthood, CR3 is exclusively expressed by microglia. Deletion of CR3 has been shown to be protective both *in vitro* and *in vivo* [8, 91]. While injection of A*β* oligomers in WT mice resulted in synapse loss, A*β* oligomers failed to induce synapse loss in of *CR3* KO mice [8], supporting the role played by the complement in synapse removal during AD.

Additionally, a new phagocytic-independent role of CR3 has been attributed to this receptor in AD models. APP AD mice with deleted CR3 expression presented less A*β* load and reduced interstitial soluble A*β* when compared with APP mice expressing normal CR3 levels [91]. Altogether, CR3 mediates phagocytosis-dependent A*β* plaque and synapse removal and also phagocytosis-independent interstitial soluble A*β* elimination. Further studies in AD models are required to further expand the key role played by CR3 in AD progression.

Finally, not only CR3 but also microglial C3aR receptors might be involved in synaptic loss in AD [14, 37]. A*β* induces astrocytic release of C3 via NF*κ*B activation, which in turn interacts with microglial C3aR to mediate pathology in AD [14, 37]. C3aR is also expressed by neurons in AD [14].

Hence, in summary, the presence of oligomeric/fibrillar A*β* at the synaptic area increases the levels of C1q and binds to it. As a result of which, the levels of C3 increase and its cleavage product iC3b binds to microglial CR3, which in turn results in synapse removal (Figure 1(c)).

## 6. CR1 and AD

CR1 is a transmembrane glycoprotein that can be found in the plasma membrane of erythrocyte and monocyte/macrophage among other cells and is involved in the phagocytosis of complement-opsonized pathogens [92].

In the periphery, CR1 is mainly expressed by erythrocytes and is involved in the clearance of complement-opsonized pathogens. It has been reported that peripheral A*β* is opsonized by the complement and captured by erythrocyte and monocyte/macrophage CR1 [21, 93]. In AD patients, capture of A*β* by erythrocytes is reduced [21, 93] and A*β* immunotherapy improves this clearance mechanism *in vitro* and in living primates [21].

Several laboratories have studied the cellular localization of CR1 within the brain. In 1996, Gasque and Morgan [38] reported that CR1 is expressed by primary human astrocytes and T193 astrocytes in vitro. Those results were later confirmed using immunohistochemistry in human tissue sections from normal and multiple sclerosis (MS) brains [38]. However, in 1999, Singhrao et al. were unable to detect CR1 in neurons, astrocytes, or microglia [94].

In 2009, a GWAS study identified single-nucleotide polymorphisms (SNP) in CR1 that were associated with late onset AD and therefore as a risk factor for the disease [77]. Further work confirmed this association [95–99], and the study of its cellular localization in the CNS intensified.

While Allen et al. and Karch et al. [100, 101] reported CR1 mRNA expression on cortical homogenates of AD brains, Holton et al. [102] found CR1 to be associated specifically with the frontal cortex white matter and cerebellum in AD samples. At cellular level, Hazrati et al. [103] showed CR1 immunoreactivity in neurons, choroid plexus, and blood cells of human origin, whereas CR1 localization in human microglia was not evident.

Based on the genetic association, expression, and function, in 2016, Fonseca et al. [104] analyzed the role of CR1 in AD. They showed the specificity of two monoclonal anti-CR1 antibodies for astrocytes both in human tissue samples and in human brain-derived astrocyte cultures. Nonspecific immunoreactivity in neurons or microglia was detected. The authors determined that the use of different antibodies with different reactivities or levels of detection could explain differences among different laboratories [104]. They also reported that neither CR1 distribution in the brain nor its binding activity correlated with AD-related CR1 polymorphisms and diagnostics. They concluded that further functional studies on peripheral CR1 on red cells might bring light on how this receptor contributes to AD.

A recent study by Johansson et al. [105] investigated the peripheral interaction of CR1 with A*β* in AD and was also unable to detect CR1 expression in the brain. By contrast and in line with previous results by their lab [93] as well as those by Fonseca et al. [104], they showed that CR1 in erythrocytes was significantly reduced in AD. CR1-mediated erythrocyte capture of circulating A*β* was also significantly reduced. Moreover, the SNP of CR1 that increases AD risk was associated with decreased CR1 in erythrocytes, while the SNP of CR1 that decreases AD risk was associated with increased CR1 in erythrocytes.

In summary, while CR1 localization in the peripheral nervous system has been strongly supported, the CR1 expression in the brain is still open to debate. If it is indeed expressed in the brain, CR1 might play a role in synapse pruning, as it is a receptor for opsonized complexes.

## 7. Beneficial versus Detrimental Role of Complement in AD

Reported data indicate that the complement system might have a dual role in AD. Some authors argue that the complement is neuroprotective. As a matter of fact, some complement factors like C3 have been reported to decrease the neuropathology in hAPP mice [43]. C3 and CR3 are involved in fibrillar A*β* phagocytosis *in vitro* [106]. Also, a study carried out using APP/C3^−/−^ mice suggested that C3 may have a beneficial role in plaque clearance and neuronal health in AD. It should be noted that such study was done using 12- and 17-month-old mice [90]. C1q has also been proven to have a neuroprotective role against A*β* toxicity both *in vitro* and in mice [107].

However, numerous researchers claim that the complement plays a detrimental role in AD. An APP C1q KO mouse model showed less glial activation and decreased synapse loss and neuronal degeneration [108]. Also, treatment with a C3aR antagonist that blocked the C3 signal in APP mice rescued cognitive impairment [14]. In another study, oligomeric A*β* failed to induce synaptic loss in C1qa KO mice, C3 KO mice, and in mice with microglia-lacking CR3 [8]. Also, inhibiting microglial CR3 reduced A*β* pathology [37]. Recent publications demonstrated that lifelong C3 deficiency partially protects against age- and A*β*-associated hippocampal synapse loss in mice [109, 110]. Although C3 deficiency resulted in less A*β* clearance and, therefore, more plaques, the mice had more synapses and better cognitive function, suggesting that the immune response to A*β* was the cause of neurodegeneration. Moreover, microglia-lacking CR3 is more efficient in degrading A*β* [91]. It has also been shown that activation of the complement system worsens tau pathology in AD models (reviewed in [111]).

All these contradictory results may be due in part to the variety of experimental models used and the different ages at which mice were studied. Also, different brain regions were analyzed. Studies that found C3 to be beneficial for A*β* clearance and neuronal death were carried out using APP [90] and hAPP [43] mice older than 10 months with well-established A*β* plaque pathology. However, some of the studies that found C1q, C3, and CR3 to be detrimental for A*β* clearance and synapse health used J20 [8] and APP [91] mice at preplaque stages.

Acute versus chronic complement activation may also be a key for understanding its dual role in AD pathology. Lian et al. [14] showed that short-term treatment of microglia with C3 or C3a promoted phagocytosis, whereas longer treatment diminished it.

As in AD, complement inhibition is protective in other neurodegenerative diseases. Increased expression of C1q by microglia [112] and in synapses has been reported in murine models of glaucoma, and its inhibition prevented synapse degeneration [113]. Moreover, C1qa was upregulated in microglia and neurons during West Nile virus infection and lack of C3 or C3aR in KO mice protected against virus-induced synaptic loss [114]. It has also been reported that complement contributes to cell and myelin damage in MS, with an upregulation of C1q, C3, and C3-activated products in patients' hippocampi [115] and plaques [116].

Finally, neuroglia express inhibitory protective molecules to prevent uncontrolled complement-mediated damage, such as CD59, complement factor H (CFH), and complement receptor-related protein-y (Crry), which mainly interfere with C3 [19, 117]. Modification of the expression of these molecules in the context of AD needs additional investigation. Whether the complement plays a beneficial or a detrimental role in AD needs further clarification.

## 8. Other Pathways Involved in Synapse Removal

Besides complement-mediated synapse removal, other pathways have been described to be involved in glia-neuron interaction that might also result in synapse elimination (reviewed in [118]). Astrocytes have been described to be implicated in activity-dependent synapse elimination via multiple EGF-like domains 10 (MEGF10) and Mer tyrosine kinase (MERTK) pathways [119]. Recently, phagocytic capacity of reactive astrocytes via ABCA1 and its pathway molecules, MEGF10 and GLUP1, after transient brain ischemia has also been reported [120]. Also, the phagocytic clearance capacity of plaque-associated reactive astrocytes of presynaptic dystrophies in an APP/PS1 AD mouse model has been demonstrated [121].

Microglial Cx3cr1 receptor might also regulate synaptic spine maintenance as reported by Paolicelli et al. [122]. Cx3cr1 KO mice showed increased levels of PSD-95 and synaptic puncta when compared to WT mice, suggesting a deficit in synaptic pruning. Also, only spontaneous vesicle release was observed. This was an indication for immature connectivity in the KO animals [122].

Additionally, a recent report [123] indicates that previously described phagocytosis of synapses by microglia might be only partial and involves specifically presynaptic terminals. The role of complement cascade in this selective phagocytosis and the requirement of glial cells need to be investigated. Additional research including knowledge about the involved signaling pathways as well as the effect of genetics, aging, disease, and brain region among others is needed to understand glia-mediated synaptic elimination.

## 9. Conclusions

Understanding the biology behind synapse loss in AD is crucial to the discovery of new and more efficient therapeutic targets, ultimately aiming to stop or reverse early stages of disease progression. *In vitro* studies showed that both intracellular [53–56] and extracellular [57, 58] A*β* are associated to synapse pathology. As a matter of fact, A*β* accumulation itself is capable of inducing synapse loss in isolated neurons. However, *in vivo* studies have demonstrated the important contribution of both microglia and astrocytes to AD-related synapse loss. Recent studies point towards a key role played by the complement pathway, specifically by C1q and/or C3 initiator proteins, as the mechanism by which glial cells modulate synapse pruning. Such mechanism may take place not only during development but also during AD-related neurodegeneration. Therefore, different effectors might be contributing to AD-associated synapse loss via different mechanisms.

The contribution of neurons and glial cells to synapse removal, both in health and during neurodegeneration, remains not well understood and needs further investigation. A number of studies suggest that there might be additional factors involved in the regulation of synaptic pruning. Thus, new knowledge on cellular and molecular contributors will shed light to the understanding of this complex machinery.

## Figures and Tables

**Table 1:** Cellular localization of complement components during development.

Cell type	Localization	Experimental model	Method	Ref.
C1q				
Neuron	Synaptic puncta, axons	*In vitro*: rat primary culture *In vivo*: C1qaKO, Tgfbr2^−/−^, C1qa^−/−^ mice	qRT-PCR, in situ hybridization, ICC, IHC, WB	[4, 5]
C3				
Unknown	Synaptic puncta	*In vivo*: C3KO mice	IHC	[4, 15]
CR3				
Microglia	Cell surface	*In vivo*: CR3KO mice	IHC	[15]

qRT-PCR: semiquantitative PCR; ICC: immunocytochemistry; IHC: immunohistochemistry; WB: Western blot.

**Table 2:** Cellular localization of complement components in adulthood and aging.

Cell type	Localization	Experimental model	Method	Ref.
C1q				
Neuron	Synaptic puncta	*In vivo*: C57BL/6 mice Human tissue	IHC	[32]
Microglia	Cellular	*In vitro*: mice primary culture *In vivo*: C57BL/6, C1qa deletion mice	qRT-PCR, IF, IHC, WB	[30–33]
Astrocyte	Exosomes	Human plasma	Immunoassay	[34]
C3				
Microglia	Cellular	*In vitro*: mice primary cultures	qRT-PCR, IF, ICC, WB	[30, 31, 35]
Astrocyte	Cellular	*In vitro*: mice primary cultures	qPCR, IF, WB, RNA-seq	[30, 35, 36]
	Exosomes	Human plasma	Immunoassay	[34]
CR3				
Microglia	—	*In vitro*: mice primary culture	qRT-PCR, IF	[31]
C3aR				
Neuron	Cellular	*In vitro*: cultured neural stem cells, rat *In vivo*: WT mice and rat	In situ hybridization, IF	[41, 42]
Microglia	Cellular	*In vivo*: WT mice Human tissue	In situ hybridization, IF, IMC	[37–39]
Astrocyte	Cellular	*In vitro*: CB193 cell line, human astrocyte cultures	IF, RT-PCR, IP	[39, 40]

qRT-PCR: semiquantitative PCR; ICC: immunocytochemistry; IHC: immunohistochemistry; WB: Western blot; IF: immunofluorescence; FACS: fluorescence-activated cell sorting; IP: immunoprecipitation.

**Table 3:** Cellular localization of complement components in Alzheimer's disease.

Cell type	Localization	Experimental model	Method	Ref.
C1q				
Neuron	Cellular	Human tissue	IHC, in situ hybridization	[88]
	Synaptic puncta	*In vivo*: J20 mice	IF	[8]
Microglia	—	*In vivo*: J20 mice	IF	[8]
Astrocyte	Cellular	*In vitro*: human primary culture *In vivo*: 5xFAD mice	ELISA, IF	[84, 89]
	Exosomes	Human plasma samples	Immunoassay	[34]
C3				
Neuron	Synaptic puncta	*In vivo*: J20 mice	IF	[8]
Microglia	—	*In vitro*: mice primary culture *In vivo*: APPswe/PS1dE9 mice	qPCR, IP, in situ hybridization	[35, 37]
Astrocyte	—	*In vitro*: mice primary culture *In vivo*: APP/TTA, APPswe/PS1dE9 mice	qPCR, in situ hybridization, IC	[30, 37]
	Cellular	*In vivo*: APP mice	IF	[124]
	—	Human tissue	qPCR, in situ hybridization	[14]
	Exosomes	Human plasma	Immunoassay	[34]
CR3				
Microglia	Cellular	*In vitro*: mice primary culture, BV2 cell line *In vivo*: J20 mice	IF, WB	[8, 91]
C3aR				
Neuron	—	*In vitro*: mice primary culture *In vivo*: APP/TTA mice	IF	[118]
Microglia	—	*In vitro*: mice primary culture *In vivo*: APPswe/PS1dE9 mice	qPCR, in situ hybridization	[37]

qRT-PCR: semiquantitative PCR; ICC: immunocytochemistry; IHC: immunohistochemistry; WB: Western blot; IF: immunofluorescence; FACS: fluorescence-activated cell sorting; IP: immunoprecipitation.

## Additional Points

*Highlights*. Amyloid-induced synapse loss in AD may occur via both neuron-autonomous mechanisms and nonautonomous mechanisms involving glial cells. Glial cells contribute chiefly to synapse pruning via the complement pathway. The complement pathway plays a key role in glia-mediated synapse pruning. Glial contribution as a source of complement components differs during development, adulthood, normal aging, and Alzheimer's disease.
