# Corrigendum to “Contribution of Neurons and Glial Cells to Complement-Mediated Synapse Removal during Development, Aging and in Alzheimer's Disease”

**DOI:** 10.1155/2019/7539620

**Published:** 2019-01-29

**Authors:** Celia Luchena, Jone Zuazo-Ibarra, Elena Alberdi, Carlos Matute, Estibaliz Capetillo-Zarate

**Affiliations:** ^1^Achucarro Basque Center for Neuroscience, CIBERNED and Departamento de Neurociencias de la Universidad del País Vasco UPV/EHU, E-48940 Leioa, Spain; ^2^IKERBASQUE, Basque Foundation for Science, 48011 Bilbao, Spain; ^3^Weill Cornell Medical College, Cornell University, New York, NY 10065, USA

In the article titled “Contribution of Neurons and Glial Cells to Complement-Mediated Synapse Removal during Development, Aging and in Alzheimer's Disease” [1], there was an error in a funding number, where “PIBA PI-2016-1-009-0016 and ELKARTEK 2016-00033” should be corrected to “PIBA PI-2016-1-0009 and ELKARTEK KK-2016-00033.” The corrected Acknowledgments section is as follows:

The authors thank Dr. Baleriola at the Achucarro Basque Center for Neuroscience (Bilbao, Spain) and Dr. Solé-Domènech and Dr. Pipalia at Weill Cornell Medical College (Cornell University, New York, USA) for the helpful and critical revision of the manuscript. This study was supported by CIBERNED and by grants from the Ministerio de Economía y Competitividad (SAF2016–75292-R), Gobierno Vasco (PIBA PI-2016-1-0009 and ELKARTEK KK-2016-00033), Ikerbasque, Basque Foundation for Science, and Universidad del País Vasco/Euskal Herriko Unibertsitatea (UPV/EHU). Jone Zuazo held a fellowship from Gobierno Vasco, and Celia Luchena from Fundación Tatiana Pérez de Guzmán el Bueno.
